# Strengthening human immunodeficiency virus surveillance through systematic data cleaning: a national retrospective validation of the human immunodeficiency virus treatment and care registry in Guyana, 2025

**DOI:** 10.1016/j.ijregi.2026.100970

**Published:** 2026-08-05

**Authors:** Tariq Jagnarine, Murvin Chalmers

**Affiliations:** National AIDS Programme Secretariat (NAPS), Ministry of Health, Georgetown, Guyana

**Keywords:** HIV surveillance, Data quality, HIV care cascade, Retention in care, Viral suppression, Guyana

## Abstract

**Background:**

Accurate human immunodeficiency virus (HIV) surveillance is essential for achieving the Joint United Nations Programme on HIV/acquired immunodeficiency syndrome (AIDS) (UNAIDS) 95-95-95 targets. Historically, Guyana’s HIV datasets contained duplicate entries, silent mortality, and unverified migration, resulting in inflated denominators and under-reported viral suppression. In 2025, the National AIDS Programme Secretariat conducted the first national HIV data-cleaning and verification exercise to establish a validated treatment cohort.

**Objectives:**

1. To verify and clean historical HIV treatment records.

2. To quantify duplicate, deceased, migrated, and defaulter cases.

3. To distinguish long- vs short-term defaulters.

4. To assess the impact on UNAIDS cascade indicators.

5. To model viral suppression gains through re-engagement.

**Methods:**

A national retrospective review of 12,824 records included deduplication, outcome verification, and cohort classification. Analytical outputs included Wilson 95% confidence intervals, chi-square distribution testing, and standardized residuals. Re-engagement modeling simulated suppression gains across 15-60% scenarios.

**Results:**

We identified 401 duplicates (3.1%), 1308 deaths (10.2%), and 454 migrations (3.5%), producing a validated eligible cohort of 10,661 people living with HIV, correcting 13.7% denominator inflation. Of eligible clients, 7268 (68.2%) were active, 2071 (19.4%) long-term defaulters, and 1322 (12.4%) short-term defaulters. Re-engagement modeling showed improvements of +2.6 percentage points at 25% return and +6.3 percentage points at 60%, indicating strong potential for accelerating the third 95.

**Conclusion:**

National data validation substantially improved surveillance precision and clarified programmatic gaps. Re-engagement of short-term defaulters is a high-impact strategy for increasing viral suppression. Guyana’s 2025 exercise provides a replicable model for improving regional HIV monitoring and advancing progress toward epidemic control.

## Highlights

• Cleaning 12,824 records verified 1308 deaths, correcting 13.7% inflation.
• Active care included 7268 clients, representing 56.7% of all records.
• Re-engaging 60% of 1322 short-term defaulters could add 6.4 percentage points.

## Introduction

Accurate, person-level human immunodeficiency virus (HIV) surveillance is fundamental to epidemic control, program accountability and the equitable allocation of health resources. In 2023, an estimated 39.9 million people were living with HIV globally, of whom approximately 30.7 million were receiving antiretroviral therapy (ART). Progress toward ending acquired immunodeficiency syndrome (AIDS) as a public health threat is assessed principally through the Joint United Nations Programme on HIV/AIDS (UNAIDS) 95-95-95 targets: 95% of people living with HIV should know their status, 95% of those diagnosed should receive sustained ART, and 95% of those receiving ART should achieve viral suppression [1]. The validity of these indicators depends not only on service coverage but also on the accuracy of the population denominators from which they are calculated.

The World Health Organization (WHO) increasingly emphasizes person-centered HIV monitoring based on longitudinal, individual-level data spanning diagnosis, treatment initiation, retention, viral-load monitoring and long-term care [2]. When appropriately governed, these systems support continuity of care, early identification of treatment interruption, differentiated service delivery and more precise measurement of national treatment outcomes. However, their reliability depends on unique patient identification, interoperability, complete outcome documentation and routine reconciliation across clinical, laboratory, pharmacy, mortality and migration data sources [2].

These requirements remain difficult to achieve in many low- and middle-income countries, where HIV information systems frequently consist of parallel paper registers, partially digitized records and non-interoperable databases. Such environments are vulnerable to duplicate registration, inconsistent identifiers, incomplete clinical histories, undocumented facility transfers, unrecorded deaths and delayed updates of treatment outcomes [3]. Systematic data cleaning is therefore more than a technical preprocessing exercise; it is an essential component of epidemiological surveillance and program governance. Evidence from Kenya demonstrated that substantial HIV-data quality problems remained undetected by automated validation procedures, underscoring the need for structured and reproducible cleaning protocols [4]. Similarly, validation of Haiti’s national HIV/AIDS case-surveillance system confirmed that deterministic and probabilistic record-matching procedures could identify duplicate records and improve the accuracy of national case estimates [5].

Misclassification of patients recorded as lost to follow-up represents a particularly important source of surveillance bias. The term frequently combines several distinct outcomes, including confirmed mortality, migration, undocumented transfer to another facility, temporary interruption of treatment, re-engagement elsewhere and prolonged disengagement from care. These outcomes have substantially different clinical and programmatic implications. An individual-patient-data meta-analysis demonstrated that patients classified as lost to follow-up often included persons who had died or transferred to other services, producing biased estimates of mortality and program retention [6]. Sampling-based tracing studies have similarly shown that routine clinic records may substantially underestimate continued engagement in care outside the original treatment facility [7]. Record linkage in South Africa further demonstrated that migration and subsequent re-engagement accounted for considerable proportions of patients initially classified as lost to follow-up [8].

Failure to resolve these classifications can distort both the numerator and denominator of national HIV cascade indicators. Duplicate records and individuals who have died or permanently migrated may inflate the population considered eligible for treatment and viral-suppression monitoring. Conversely, undocumented transfers and re-engagement at other facilities may cause active patients to be incorrectly classified as disengaged. These errors can artificially lower reported ART coverage and viral-suppression performance, misdirect tracing resources and obscure the true distribution of treatment interruption. Distinguishing short-term interruption from long-term disengagement is especially important because recently disengaged patients may represent a high-yield population for targeted re-engagement and rapid restoration of viral suppression.

These challenges are particularly relevant to Caribbean health systems, where relatively small national cohorts coexist with cross-border mobility, decentralized service delivery and varying levels of health-information digitization. In Guyana, the national HIV treatment and care registry developed through successive periods of paper-based recording, facility-level databases and progressive electronic integration. Although these systems supported the expansion of HIV services, the accumulation of historical records created risks of duplication, silent mortality, undocumented migration and unresolved treatment outcomes. Before the present exercise, Guyana had not undertaken a comprehensive national validation of the HIV treatment and care registry since the expansion of ART services.

In 2025, the National AIDS Programme Secretariat conducted a systematic national cleaning and retrospective validation of Guyana’s HIV treatment and care registry. This study aimed to establish verified clinical-status classifications; quantify duplicate records, mortality and migration; distinguish short-term from long-term treatment disengagement; determine the effect of denominator correction on national HIV cascade indicators; and model potential improvements in viral suppression through targeted re-engagement. By examining data cleaning as both a surveillance intervention and a mechanism for improving program performance, this study provides evidence relevant to Guyana and other resource-constrained Caribbean health systems undergoing digital transformation.

## Study objectives

### General objective

To evaluate how systematic cleaning and validation of Guyana’s national HIV treatment and care registry improved the accuracy of patient-status classifications, national HIV cascade denominators and projected viral-suppression outcomes during the 2025 validation exercise.

### Specific objectives

1. Validate all 12,824 registry records using identifier matching, duplicate review and verification of mortality, migration and treatment status during the 2025 data-cleaning exercise.
2. Classify each validated record into one predefined outcome category: duplicate, deceased, migrated, active in care, short-term defaulter or long-term defaulter.
3. Quantify the number, proportion and 95% confidence interval (CI) of records within each clinical-status category.
4. Calculate the absolute and relative changes in the national HIV treatment denominator following the removal of duplicate, deceased and migrated records.
5. Compare HIV treatment and viral-suppression indicators before and after denominator correction to determine the effect of data cleaning on national cascade estimates.
6. Determine the number and proportion of short-term defaulters eligible for targeted tracing and re-engagement interventions.
7. Model the expected percentage-point improvement in national viral suppression under 15%, 25%, 40% and 60% short-term-defaulter re-engagement scenarios.

## Research questions

### Primary research question

How does systematic cleaning and validation of Guyana’s 2025 national HIV treatment and care registry affect patient-status classification, the national treatment denominator and projected viral-suppression performance?

### Specific research questions

1. What are the number, proportion and 95% CI of records classified as duplicate, deceased, migrated, active in care, short-term defaulter or long-term defaulter?
2. What proportion of the original 12,824 registry records represents duplicates, verified deaths or confirmed migration?
3. What absolute and relative changes occur in the national treatment-eligible denominator after excluding duplicate, deceased and migrated records?
4. What proportion of disengaged patients are classified as short-term defaulters compared with long-term defaulters?
5. Which patient-status categories contribute most substantially to discrepancies between the historical and validated registry?
6. How does denominator correction affect estimated national ART coverage and viral-suppression indicators?
7. What percentage-point improvements in national viral suppression are projected if 15%, 25%, 40% or 60% of short-term defaulters are successfully re-engaged?
8. What proportion of short-term defaulters must be re-engaged to produce a clinically and programmatically meaningful improvement in national viral suppression?

## Literature review

### HIV surveillance and data quality

The global HIV response increasingly depends on the availability of accurate, timely and person-level surveillance data. The UNAIDS 95-95-95 framework requires countries to measure progression from diagnosis to ART and viral suppression using clearly defined and internally consistent populations [1]. Errors in patient-level records can therefore affect national estimates, apparent program performance and the interpretation of progress toward epidemic control.

WHO recommends person-centered monitoring systems capable of following individuals longitudinally across HIV testing, treatment initiation, pharmacy refills, viral-load testing, treatment interruption, transfer and re-engagement [2]. These systems should incorporate unique patient identifiers, standardized data elements, interoperability and appropriate privacy protections. Sustainable surveillance also requires national programs to transition from periodic aggregate reporting toward routinely updated patient-level information that can guide both clinical care and public health interventions [3].

Data quality is multidimensional and includes accuracy, completeness, consistency, timeliness, validity and uniqueness. A registry may be complete in terms of record volume while remaining epidemiologically inaccurate because of duplicate entries, outdated clinical outcomes or unresolved transfers. WHO therefore recommends institutionalized data-quality assessments followed by corrective action plans, national adjustment of ART estimates and routine monitoring of remedial interventions [9]. This positions data cleaning as a continuous component of program governance rather than a single retrospective activity.

### Data-quality challenges in HIV registries

HIV registries in resource-constrained settings commonly develop through successive phases of paper-based reporting, standalone facility databases and partially integrated electronic systems. During these transitions, parallel records may be maintained for the same patient, particularly when facilities lack a universal identifier or cannot exchange information. Misspellings, changes in names, incomplete dates of birth, inconsistent demographic information and the reuse of temporary identifiers further complicate record linkage.

Systematic cleaning of HIV indicator data in Kenya identified errors that had not been detected through routine automated validation, demonstrating that electronic reporting alone does not guarantee reliable data [4]. A multinational survey of ART programs similarly found persistent challenges involving incomplete records, inconsistent data entry and the classification of patients lost to follow-up, even where electronic medical records had been introduced [10]. These findings indicate that information-system digitization must be accompanied by governance standards, trained personnel, routine audits and procedures for resolving discordant records.

Duplicate registration is particularly important because it can inflate the apparent number of diagnosed individuals or persons receiving treatment. Duplication may occur when a patient transfers without documentation, registers under a different name, returns after prolonged treatment interruption or seeks care at multiple facilities. Validation of Haiti’s national HIV surveillance system demonstrated that combining deterministic and probabilistic matching with expert review improved duplicate identification and national case ascertainment [5]. However, probabilistic matching must be carefully governed because overly broad algorithms may incorrectly merge records belonging to different individuals, while overly restrictive algorithms may fail to identify true duplicates.

### Digital-health systems and national HIV surveillance

Electronic medical records offer substantial advantages for longitudinal HIV surveillance, including automated appointment monitoring, treatment-history reconstruction, viral-load tracking and identification of patients requiring clinical follow-up. Nevertheless, digital systems reproduce the quality of the information entered into them and may introduce additional errors through delayed entry, transcription from paper files, software downtime and inconsistent use across facilities.

An assessment of 3467 HIV records from 50 health facilities in Rwanda found generally high completeness for many data elements but persistent deficiencies in viral-load results, advanced ART regimens and the concordance of clinical dates between paper and electronic records [11]. This demonstrates that clinically important variables may remain incomplete even when the overall electronic record appears satisfactory. Because viral-load outcomes are central to the third UNAIDS 95, missing or incorrectly linked laboratory results can materially distort national suppression estimates.

National data warehouses can improve surveillance by integrating individual-level data from multiple facility-based electronic systems. Kenya’s national HIV data warehouse expanded to include data from 1516 facilities across 41 of the country’s 47 counties, demonstrating the feasibility of consolidating longitudinal HIV information at scale [12]. However, centralized repositories require robust deduplication, standardized identifiers, data-transfer protocols and routine reconciliation with laboratory, pharmacy, mortality and migration systems. Without these safeguards, centralization may aggregate pre-existing errors rather than correct them.

### Misclassification of loss to follow-up

Loss to follow-up is not a single clinical outcome. It is an administrative classification applied when a patient has not returned within a specified period and their subsequent status is unknown. Individuals categorized as lost to follow-up may have died, migrated, transferred silently to another facility, temporarily interrupted treatment or remained in care outside the reporting system.

An individual-patient-data meta-analysis found that tracing patients classified as lost to follow-up frequently identified undocumented mortality and transfers, demonstrating that routine clinic records may produce biased retention and mortality estimates [6]. Sampling-based tracing has similarly shown that many patients considered disengaged from their original facility remain connected to care elsewhere [7]. In South Africa, record linkage identified substantial migration and re-engagement among patients initially classified as lost to follow-up [8].

These findings have important implications for national surveillance. If deceased or permanently migrated individuals remain in the treatment denominator, ART coverage and viral suppression may be underestimated. Conversely, if undocumented transfers are classified as defaulters, programs may overestimate disengagement and direct tracing resources toward individuals already receiving care elsewhere. Reliable outcome verification therefore requires linkage with civil-registration, facility-transfer, pharmacy, laboratory and community-tracing data.

The duration of disengagement is also programmatically important. Patients who recently interrupted ART are generally more likely to be located and re-engaged than those whose last documented contact occurred many years earlier. Combining recent and historical defaulters into one category can therefore conceal differences in traceability, mortality risk and intervention potential. Stratifying disengagement by duration provides a more operationally useful basis for prioritizing re-engagement resources.

### Re-engagement and viral suppression

Treatment interruption increases the likelihood of viral rebound, disease progression, onward transmission and the development of drug resistance. Re-engagement is therefore necessary not only to improve retention statistics but also to restore treatment continuity and population-level viral suppression.

A systematic review and meta-analysis found that approximately 39% of patients initially classified as lost to follow-up returned to their original clinic following re-engagement activities. Among individuals confirmed to be alive and out of care, 58% returned after telephone or in-person contact. Approximately 64% were subsequently retained in care, while 56% achieved viral suppression [13]. However, substantial heterogeneity was observed across settings, intervention designs and definitions of loss to follow-up.

Evidence also supports the use of patient navigation, appointment reminders, psychosocial support, transportation assistance and data-to-care interventions. A meta-analysis of 42 interventions found significant improvements in re-engagement, retention and viral suppression, with pooled odds ratios of 1.79, 2.01 and 2.50, respectively [14]. Nevertheless, most included studies were conducted in the United States, limiting direct generalizability to Caribbean health systems. Local implementation should therefore account for transportation barriers, migration, stigma, confidentiality, mobile-phone access, health literacy and facility capacity.

These findings support scenario modeling as a planning tool for estimating the potential effect of re-engagement on national viral suppression. Such models should not be interpreted as causal predictions because successful suppression after return to care depends on adherence, drug resistance, regimen availability, psychosocial circumstances and continued retention. Scenario estimates are most useful for program planning when accompanied by sensitivity analyses and subsequent operational validation.

### Caribbean evidence and the Guyana knowledge gap

Published evidence on national HIV registry validation in the Caribbean remains limited. Haiti has provided important regional experience in national case-based surveillance, deduplication and the use of electronic medical records for quality improvement [5]. The development of Haiti’s iSanté system demonstrated how individual-level information could support national monitoring, quality-management activities and real-time identification of care gaps [15]. However, these experiences are not automatically transferable to other Caribbean countries because of differences in population size, migration patterns, facility networks and digital-health infrastructure.

Guyana’s HIV information system developed through a combination of paper registers, facility-level electronic records and progressive national digitization. This history created the potential for duplicate registration, unresolved mortality, undocumented migration, silent transfer and inconsistent treatment-status classification. Despite the importance of these issues, there was limited empirical evidence quantifying how historical data inaccuracies affected Guyana’s national HIV treatment denominator and viral-suppression indicators.

The present study addresses this gap by undertaking a national retrospective validation of the HIV treatment and care registry. Its principal contribution is the integration of record deduplication, mortality and migration verification, duration-based classification of treatment disengagement, denominator correction and viral-suppression scenario modeling within one national analysis. This approach extends the literature beyond descriptions of data-quality problems by demonstrating how systematic registry validation can produce directly actionable information for surveillance, re-engagement planning and national progress toward the UNAIDS targets.

## Methodology

### Study design, data source and population

A retrospective, census-based validation of Guyana’s national HIV treatment and care registry was conducted in 2025. The study included all 12,824 records contained in the registry from national HIV treatment scale-up through the data-lock date of January 31**,** 2025. Because the complete national registry was evaluated, no sampling procedure or sample-size calculation was required. The unit of analysis was the individual registry record before deduplication and the verified person after record reconciliation.

### Data-cleaning and validation procedures

Data cleaning was undertaken sequentially to standardize identifiers, identify possible duplicate records, validate reported outcomes and assign each person to one mutually exclusive status category. Matching variables included, where available, the unique patient or clinic identifier, full name, sex, date of birth or age, treatment facility, address or contact information and documented treatment history.

Potential duplicates were initially identified through exact deterministic matching of unique identifiers and combinations of demographic variables. Phonetic similarity review was subsequently applied to names with spelling variations, transcription errors or inconsistent formatting. Records were not classified as duplicates solely because of name similarity. Potential matches required corroboration through additional demographic, facility or clinical information and were manually reviewed before removal. This process identified 401 duplicate records.

Following deduplication, the remaining records were assessed according to the following hierarchical decision sequence:

1. Duplicate record
2. Verified death
3. Confirmed migration
4. Active in care
5. Short-term treatment interruption
6. Long-term disengagement

Deaths were verified through available clinical reports, hospital records, facility audits or documented community tracing. Migration was confirmed through facility notification, transfer information or field follow-up. Applying the categories sequentially ensured that each person contributed to only one final outcome.

Records that could not be resolved through automated matching underwent manual verification by the national data-cleaning team. As this was an operational surveillance exercise rather than a blinded diagnostic-validation study, formal independent dual review and inter-reviewer agreement statistics were not undertaken.

### Operational definitions

**Table:** 

Classification	Operational definition
Duplicate record	More than one record determined to represent the same person following identifier matching and manual verification
Verified death	Death confirmed through clinical, hospital, facility or documented community sources
Confirmed migration	Documented relocation outside the national treatment-reporting population
Active in care	Documented clinical, pharmacy or program contact within 30 days of the data-lock date
Short-term treatment interruption	No documented contact for more than 30 days but less than seven years
Long-term disengagement	No documented contact for seven years or longer

The seven-year threshold was an operational program classification used to distinguish potentially traceable treatment interruption from prolonged historical disengagement. It should not be interpreted as a universally validated clinical definition of loss to follow-up.

### Study outcomes

The primary outcome was the absolute and relative change in the national treatment-eligible denominator following the removal of duplicate records, verified deaths and confirmed migration.

Secondary outcomes included:

• Number and proportion of records in each validated category
• Prevalence of duplicate registration
• Proportion of verified mortality and migration
• Proportion active in HIV care
• Distribution of short-term and long-term treatment disengagement
• Number of short-term disengaged patients potentially eligible for re-engagement
• Projected increase in population-level viral suppression under alternative re-engagement scenarios

### Denominator recalculation

The validated treatment-eligible denominator was calculated as:

Dv=N−(Nd+Nm+Nx)

Where:

• *Dv* = validated treatment-eligible denominator
• *N* = total number of registry records
• *N_d_* = confirmed duplicate records
• *N_m_* = verified deaths
• *N_x_* = confirmed migration

The absolute denominator reduction was calculated as the difference between the original and validated denominators. The relative reduction was calculated as:

$$\text{Relative}\;\text{reduction}=\frac{N-Dv}{N}\;\times 100$$

Denominator reduction attributable specifically to mortality and migration was calculated separately from the total reduction that also included duplicate records.

### Viral-suppression scenario modeling

Scenario modeling estimated the potential increase in population-level viral suppression if selected proportions of short-term treatment interrupters were successfully re-engaged. Four re-engagement scenarios were evaluated: 15%, 25%, 40% and 60%.

The principal model assumed that 85% of successfully re-engaged individuals would subsequently achieve viral suppression. The number expected to achieve suppression under each scenario was calculated as:

Nvs=Ns×R×0.85

Where:

• *N_vs_* = additional individuals expected to achieve viral suppression
• *N_s_* = number of short-term treatment interrupters
• *R* = proportion re-engaged under each scenario
• 0.85 = assumed probability of suppression following re-engagement

The projected percentage-point increase was calculated as:

$$\Delta VS\;=\;\frac{Ns\times R\times 0.85\;}{Dv}\;\times 100$$

These projections represent potential improvements in population-level suppression coverage among the validated cohort. They should not be described as direct changes in the third UNAIDS 95 unless the number receiving ART and the number with documented viral suppression are used as the appropriate numerator and denominator.

### Statistical analysis

Categorical outcomes were summarized using frequencies and proportions. Wilson 95% CIs were calculated for the principal proportions. Results were reported using both the original registry denominator of 12,824 records and the validated treatment-eligible denominator of 10,661 persons.

The validated denominator was calculated as:

Dv=N−(Nd+Nm+Nx)

where *N* represents the original registry, *N_d_* duplicate records, *N_m_* verified deaths, and *N_x_* confirmed migration.

No hypothesis test was undertaken to compare the observed categories with a uniform distribution because equal expected frequencies had no epidemiological or program-based justification. Accordingly, the chi-square goodness-of-fit test, standardized residuals and associated *P*-values were excluded from the revised analysis.

Re-engagement modeling was conducted for 15%, 25%, 40% and 60% of the 1322 individuals experiencing short-term treatment interruption. The principal analysis assumed that 85% of successfully re-engaged individuals would achieve viral suppression:

Nvs=Ns×R×0.85

$$\Delta VS\;=\;\frac{Ns\times R\times 0.85\;}{Dv}\;\times 100$$

Sensitivity analyses applied conservative, intermediate and optimistic post-re-engagement suppression probabilities of 56%, 70% and 85%, respectively. These scenarios were deterministic projections; therefore, *P*-values were neither applicable nor reported. Analyses were conducted using R version 4.3 [16], and reporting was guided by the Strengthening the Reporting of Observational Studies in Epidemiology guidelines [17].

### Data quality assurance

Potential duplicates and discordant outcomes underwent manual verification before final classification. The cleaning process preserved an audit trail documenting the original record, reason for review, evidence examined, final classification and action taken.

Only confirmed duplicate records, verified deaths and confirmed migration were removed from the treatment-eligible denominator. Records with uncertain outcomes were not automatically classified as deceased or migrated.

### Ethical considerations

Ethical approval was obtained from the Institutional Review Board, Guyana, under reference number 85/2024. The analysis used de-identified routine program data. Direct personal identifiers were excluded from the analytical dataset, and access was restricted to authorized study and program personnel. Results were reported in aggregate form to prevent identification of individual patients.

### Reporting standard

The study should be reported in accordance with the Strengthening the Reporting of Observational Studies in Epidemiology guidance and applicable recommendations for studies using routinely collected health data.

## Results

### Registry validation and outcome classification

A total of 12,824 records were included in the national HIV registry validation. Following sequential review, 401 records were confirmed as duplicates, representing 3.1% (95% CI: 2.8-3.4%) of the original registry. Among the remaining unique records, 1308 persons were confirmed deceased and 454 were confirmed to have migrated. These represented 10.2% (95% CI: 9.7-10.7%) and 3.5% (95% CI: 3.2-3.9%) of the original registry, respectively.

Of the original registry, 7268 persons were classified as active in care, representing 56.7% (95% CI: 55.8-57.5%). Long-term disengagement accounted for 2071 persons, or 16.1% (95% CI: 15.5-16.8%), while 1322 persons, or 10.3% (95% CI: 9.8-10.8%), were classified as experiencing short-term treatment interruption. The six mutually exclusive classifications accounted for all 12,824 registry records, as presented in Table 1.

**Table 1:** Validated distribution of national HIV registry records.

Patient-status category	Number	Percentage of original registry	95% CI
Duplicate records	401	3.1%	2.8-3.4%
Verified deaths	1308	10.2%	9.7-10.7%
Confirmed migration	454	3.5%	3.2-3.9%
Active in care	7268	56.7%	55.8-57.5%
Long-term disengagement	2071	16.1%	15.5-16.8%
Short-term treatment interruption	1322	10.3%	9.8-10.8%
**Total**	**12,824**	**100.0%**	—

Abbreviation: CI, confidence interval; HIV, human immunodeficiency virus.

### Derivation of the validated treatment-eligible denominator

The sequential derivation of the validated treatment-eligible cohort is illustrated in Figure 1. Removal of 401 duplicate records reduced the registry from 12,824 to 12,423 unique records. Exclusion of 1308 verified deaths produced 11,115 records. The subsequent exclusion of 454 persons with confirmed migration resulted in a final validated treatment-eligible denominator of 10,661 persons.

**Figure 1:**
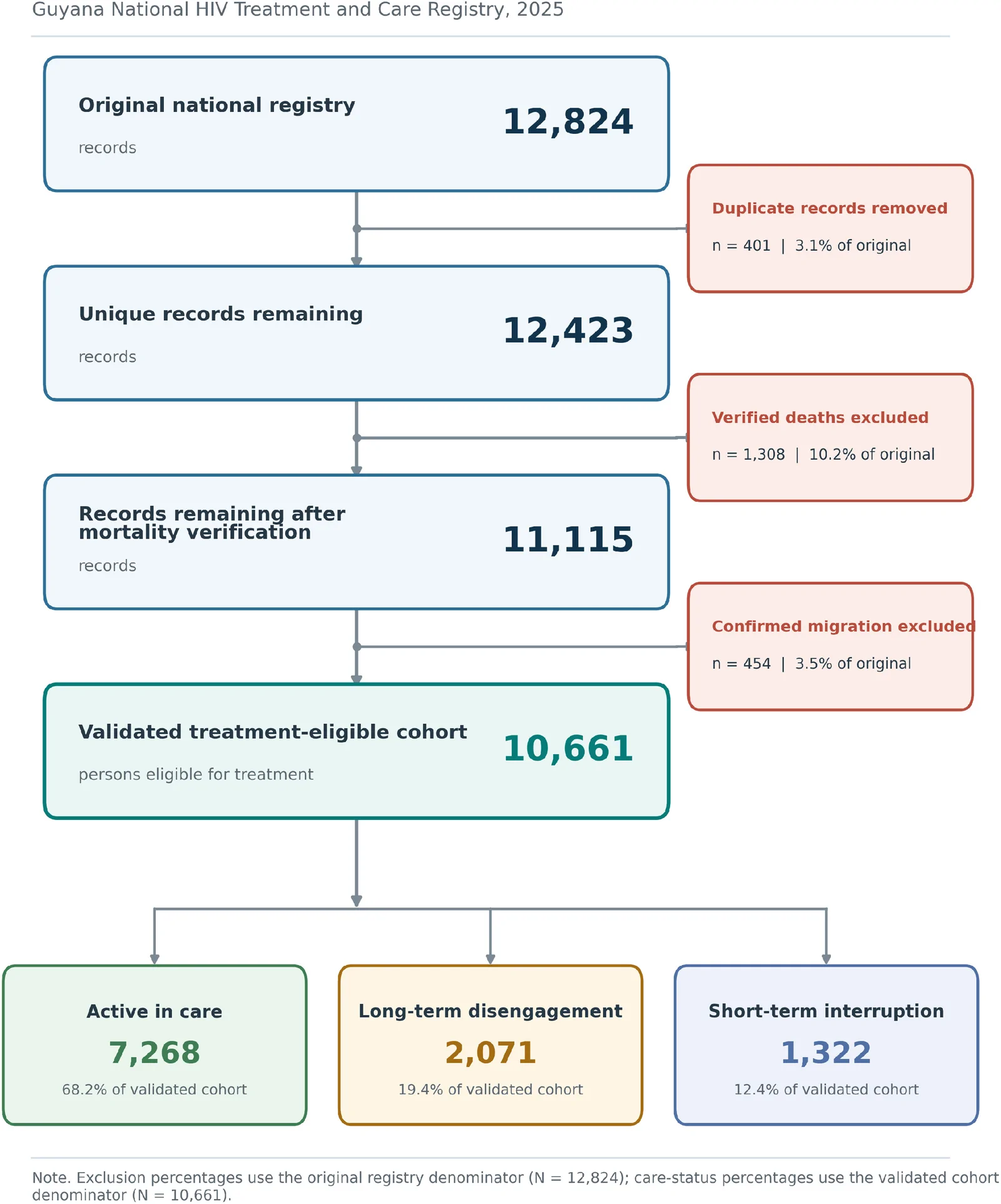
Derivation of the validated treatment-eligible cohort. • Original national registry: 12,824 records • Duplicate records removed: 401 • Unique records remaining: 12,423 • Verified deaths excluded: 1308 • Records remaining: 11,115 • Confirmed migration excluded: 454 • Validated treatment-eligible cohort: 10,661 o Active in care: 7268 o Long-term disengagement: 2071 o Short-term treatment interruption: 1322

Verified deaths and confirmed migration collectively accounted for 1762 exclusions, equivalent to 13.7% (95% CI: 13.2-14.3%) of the original registry. When duplicate records were also included, the complete denominator correction was 2163 records, representing 16.9% (95% CI: 16.2-17.5%) of the original registry. The sequential effect of each validation stage is presented in Table 2.

**Table 2:** Effect of validation on the national treatment denominator.

Validation stage	Records remaining	Records excluded	Percentage of original registry excluded
Original registry	12,824	—	—
After deduplication	12,423	401	3.1%
After mortality verification	11,115	1308	10.2%
After migration verification	10,661	454	3.5%
**Total correction**	**10,661**	**2163**	**16.9%**

### Treatment status within the validated cohort

Within the validated treatment-eligible cohort of 10,661 persons, 7268 were active in care, representing 68.2% (95% CI: 67.3-69.1%). Long-term disengagement accounted for 2071 persons, or 19.4% (95% CI: 18.7-20.2%), while short-term treatment interruption accounted for 1322 persons, or 12.4% (95% CI: 11.8-13.0%).

Overall, 3393 persons were classified as experiencing either short-term treatment interruption or long-term disengagement. This represented 31.8% (95% CI: 30.9-32.7%) of the validated cohort. Persons experiencing short-term interruption constituted 39.0% of all disengaged individuals. The treatment-status distribution within the validated cohort is summarized in Table 3.

**Table 3:** Treatment status within the validated cohort.

Treatment status	Number	Percentage of validated cohort
Active in care	7268	68.2%
Long-term disengagement	2071	19.4%
Short-term treatment interruption	1322	12.4%
**Total validated cohort**	**10,661**	**100.0%**

### Primary re-engagement scenario modeling

Deterministic scenario modeling was undertaken among the 1322 persons classified as experiencing short-term treatment interruption. Four re-engagement scenarios—15%, 25%, 40% and 60%—were examined. The primary model assumed that 85% of successfully re-engaged persons would subsequently achieve viral suppression.

Under the 15% scenario, approximately 198 persons would be re-engaged, of whom 169 were projected to achieve viral suppression. This corresponded to a projected population-level increase of 1.6 percentage points relative to the validated treatment-eligible denominator.

Re-engaging 25% of the short-term interruption group would involve approximately 331 persons and was projected to result in 281 additional persons achieving viral suppression, equivalent to a 2.6-percentage-point increase. Under the 40% scenario, approximately 529 persons would be re-engaged, with 449 projected to achieve viral suppression, producing an estimated increase of 4.2 percentage points. The 60% scenario involved approximately 793 persons, of whom 674 were projected to achieve viral suppression, corresponding to a 6.3-percentage-point increase. The primary scenario results are presented in Table 4.

**Table 4:** Projected effect of short-term re-engagement.

Re-engagement scenario	Individuals re-engaged	Expected to achieve suppression	Potential increase
15%	198	169	1.6 percentage points
25%	331	281	2.6 percentage points
40%	529	449	4.2 percentage points
60%	793	674	6.3 percentage points

### Sensitivity analysis

Sensitivity analysis assessed conservative, intermediate and optimistic post-re-engagement viral-suppression probabilities of 56%, 70% and 85%, respectively. The projected population-level gain increased with both the proportion of persons re-engaged and the assumed probability of achieving viral suppression, as shown in Table 5.

**Table 5:** Sensitivity analysis of projected population-level viral-suppression gains.

Re-engagement scenario	Persons re-engaged	56% suppression	70% suppression	85% suppression
15%	198	111; +1.0 points	139; +1.3 points	169; +1.6 points
25%	331	185; +1.7 points	231; +2.2 points	281; +2.6 points
40%	529	296; +2.8 points	370; +3.5 points	449; +4.2 points
60%	793	444; +4.2 points	555; +5.2 points	674; +6.3 points

Values represent the projected number achieving viral suppression and the corresponding absolute percentage-point increase relative to the validated denominator of 10,661. These are deterministic projections rather than observed outcomes.

Under the 15% re-engagement scenario, the projected increase ranged from 1.0 percentage point at 56% suppression to 1.6 percentage points at 85% suppression. Under the 25% scenario, the projected gain ranged from 1.7 to 2.6 percentage points. Re-engagement of 40% produced projected gains ranging from 2.8 to 4.2 percentage points, while the 60% scenario produced gains ranging from 4.2 to 6.3 percentage points.

At the intermediate 70% assumption, the projected gains were 1.3, 2.2, 3.5 and 5.2 percentage points across the 15%, 25%, 40% and 60% re-engagement scenarios, respectively. These findings demonstrate that the projected program effect is materially dependent on viral-suppression outcomes following return to care.

The projections reported in Table 4, Table 5 represent deterministic estimates rather than observed clinical outcomes. They estimate potential incremental changes in population-level suppression coverage relative to the validated denominator of 10,661 and do not constitute a direct recalculation of the third UNAIDS 95. Accordingly, inferential *P*-values and CIs were not calculated for the modeling scenarios.

### Viral-suppression scenario modeling

Scenario modeling evaluated the potential effect of re-engaging the 1322 individuals experiencing short-term treatment interruption. Assuming that 85% of successfully re-engaged individuals achieved viral suppression, re-engaging 15% of this population could result in approximately 169 additional virally suppressed individuals and a 1.6-percentage-point increase in population-level suppression coverage.

Under the 25% scenario, approximately 331 individuals would be re-engaged, of whom 281 would be expected to achieve suppression, producing a 2.6-percentage-point increase. Re-engagement of 40% could add approximately 449 suppressed individuals and improve coverage by 4.2 percentage points. The 60% scenario could result in approximately 674 additional suppressed individuals and a 6.3-percentage-point improvement. (See Table 4).

These projections represent potential improvements in population-level viral-suppression coverage within the validated cohort. They should not be interpreted as recalculated third-95 estimates because the baseline number of individuals on ART with documented viral suppression was not included in the modeling dataset.

## Discussion

### Principal findings

This national registry-validation study demonstrated that systematic data cleaning materially changed the population denominator available for monitoring HIV treatment and care in Guyana. Of 12,824 historical registry records, 401 duplicates, 1308 verified deaths and 454 confirmed migrations were identified. These corrections produced a validated treatment-eligible cohort of 10,661 persons, representing a total denominator reduction of 16.9%. Verified deaths and migration accounted for 13.7% of the original registry, while duplicate records accounted for the remaining correction.

Within the validated cohort, 68.2% were active in care, 19.4% were classified as experiencing long-term disengagement and 12.4% were classified as experiencing short-term treatment interruption. Overall, 31.8% of the validated treatment-eligible cohort had some degree of disengagement at the data-lock date. Short-term interruption represented 39.0% of all persons classified as disengaged, identifying a substantial population that may benefit from timely status verification and re-engagement interventions.

The findings demonstrate an important distinction between a historical registry and a current treatment-eligible population. Without systematic validation, duplicate records, unrecorded deaths and undocumented migration remain embedded in national datasets and may distort program denominators, resource planning and interpretation of progress toward global HIV targets [1,2].

### Significance of denominator correction

The 16.9% total denominator correction is epidemiologically and programmatically important. An inflated denominator may make ART coverage and population-level viral-suppression coverage appear lower than they are, potentially misrepresenting program performance and the number of people requiring intervention. Conversely, removing records without adequate verification could underestimate the population requiring care. The sequential approach used in this study—deduplication followed by verification of deaths, migration and treatment status—therefore provided a more defensible denominator while preserving records for which exclusion could not be substantiated.

The identification of 401 duplicate records, representing 3.1% of the historical registry, illustrates the vulnerability of hybrid paper–electronic systems to repeated registration, identifier inconsistencies and transfers between treatment facilities. Similar challenges have been documented in Haiti, where deterministic and probabilistic matching improved the identification of duplicate HIV surveillance reports [5]. Deduplication is therefore not solely a technical database procedure; it directly affects the validity of case counts, treatment denominators and longitudinal patient monitoring.

The 1308 verified deaths represented cumulative historical deaths identified during the validation exercise. This proportion should not be interpreted as an annual mortality rate because the analysis did not include a defined person-time denominator or dates of death for rate estimation. Nevertheless, the number of deaths remaining in the active registry demonstrates the importance of routine linkage between HIV surveillance systems, health-facility records and civil registration and vital statistics systems. Evidence from African treatment programs similarly shows that people classified as lost to follow-up may have died, transferred silently or continued treatment elsewhere [[6], [7], [8]].

Confirmed migration accounted for 454 records. Although these individuals were appropriately excluded from the national treatment-eligible denominator, migration remains difficult to ascertain in settings characterized by cross-border mobility and incomplete transfer documentation. Some individuals classified as disengaged may therefore have transferred to another facility or resumed treatment outside Guyana. Improved regional and facility-level transfer documentation would further reduce this uncertainty.

### Treatment disengagement and program interpretation

After denominator validation, 7268 of 10,661 treatment-eligible persons were active in care. However, the remaining 3393 persons were classified as experiencing short-term interruption or long-term disengagement. This 31.8% represents cumulative status at the data-lock date and should not be interpreted as an annual treatment-interruption rate.

The distinction between short-term interruption and long-term disengagement improves the operational usefulness of the registry. Persons with relatively recent treatment interruption are generally more appropriate for rapid tracing, appointment support, medication restart and differentiated service delivery. In contrast, records showing prolonged disengagement may require more extensive verification of vital status, migration, silent transfer and continued residence before intensive re-engagement resources are deployed.

Nevertheless, the operational category of short-term interruption—more than 30 days but less than seven years—is broad. It potentially combines people with very different probabilities of return to care. Future surveillance cycles should consider shorter, mutually exclusive intervals, such as 31-89 days, 90-179 days, 6-11 months, 1-2 years and more than 2 years. Such stratification would support more timely and proportionate interventions and improve comparability with internationally used definitions of interruption in treatment.

### Re-engagement and projected viral-suppression gains

The scenario analysis identified potentially meaningful population-level gains from re-engaging persons experiencing short-term treatment interruption. Under the primary assumption that 85% of successfully re-engaged persons would achieve viral suppression, re-engagement of 25% of this population was projected to result in 281 additional persons achieving suppression, equivalent to a 2.6-percentage-point population-level increase. Re-engagement of 60% was projected to result in 674 additional persons achieving suppression, corresponding to a 6.3-percentage-point increase, as presented in Table 4.

These findings should be interpreted as conditional planning scenarios rather than predicted program outcomes. Successful re-engagement requires several sequential steps: locating the individual, confirming that the person is alive and resident, establishing that the person is currently out of care, facilitating return to treatment, supporting ART continuation and obtaining a documented viral-load result. Attrition at any stage would reduce the eventual suppression gain.

The sensitivity analysis in Table 5 further demonstrates that the projected effect is materially influenced by the probability of achieving viral suppression after re-engagement. Under the conservative 56% suppression assumption, projected gains ranged from 1.0 to 4.2 percentage points across the four re-engagement scenarios. Under the optimistic 85% assumption, the corresponding range was 1.6 to 6.3 percentage points. At the intermediate 70% assumption, gains ranged from 1.3 to 5.2 percentage points.

This variation reinforces the need to accompany tracing activities with adherence support, rapid ART restart, differentiated refill options, viral-load monitoring and continued retention services. Evidence from systematic reviews indicates that patient navigation, appointment support, psychosocial assistance, transportation support and data-to-care interventions can improve re-engagement and subsequent HIV care outcomes [13,14]. However, the effectiveness of these interventions varies across settings, populations and implementation models.

### Implications for UNAIDS cascade measurement

The study primarily improved denominator validity; it did not directly demonstrate improved ART coverage or viral suppression. A complete, temporally aligned numerator for all persons receiving ART and all persons with documented viral suppression was not available. Consequently, the second and third UNAIDS 95 indicators could not be directly recalculated.

The projected percentage-point gains in Table 4, Table 5 represent potential changes in population-level suppression coverage relative to the validated denominator of 10,661. They should not be interpreted as observed improvements in the third UNAIDS 95, which requires the number of people with viral suppression to be assessed against an appropriately defined ART denominator.

The next analytical step should link the validated registry with current pharmacy-dispensing, ART, laboratory and viral-load records. This would permit direct recalculation of the proportion receiving ART and the proportion of persons receiving ART who have achieved viral suppression. WHO guidance emphasizes the importance of person-centered monitoring, unique identifiers, interoperable systems and routine reconciliation across HIV service data sources [2,9].

### Digital-health and health-system implications

Registry digitization alone does not guarantee accurate surveillance. Electronic systems may reproduce or amplify errors originating from incomplete paper records, inconsistent identifiers, delayed entry and weak transfer documentation. Studies of electronic medical records in resource-limited settings have shown that data quality depends on trained personnel, reliable system availability, clearly defined workflows, routine quality assessment and appropriate clinical alerts [10,11]. National data warehouses may strengthen HIV programming, but their effectiveness depends on governance, interoperability and the consistent use of person-level identifiers [12].

Guyana’s registry-validation exercise therefore provides a foundation for a continuous data-quality system rather than a one-time correction. Priority measures should include lifelong unique patient identifiers, routine duplicate detection, electronic alerts for missed appointments, quarterly facility reconciliation and periodic linkage with mortality, pharmacy and laboratory systems. Experiences from Haiti further demonstrate that electronic medical records are most effective when integrated with national quality-management systems and facility-level improvement processes [15].

### Regional relevance

The challenges identified in Guyana are relevant to other Caribbean and Latin American settings using hybrid paper and electronic surveillance systems. Population mobility, fragmented records, delayed mortality reporting and incomplete interoperability can affect both national case counts and treatment-cascade measurement. The Guyana experience demonstrates that national registry validation is feasible and can identify substantial discrepancies that are not visible through routine aggregate reporting.

However, the magnitude of correction observed in this study should not be assumed to apply directly to other countries. Differences in registry maturity, unique-identifier coverage, migration, mortality registration and electronic-system integration will affect the type and extent of data-quality problems. The principal transferable lesson is the value of a transparent, sequential and verifiable validation process.

### Strengths and interpretive considerations

A major strength of this study was its national census-based design, which included all 12,824 records rather than a sample of facilities or patients. The sequential validation process produced mutually exclusive outcome classifications and clearly distinguished the historical registry denominator from the validated treatment-eligible population. The use of multiple sources to verify deaths and migration further strengthened classification accuracy. Sensitivity analysis also provided a more transparent assessment of uncertainty than reliance on a single suppression assumption.

These strengths should be considered alongside the limitations of historical documentation, incomplete linkage with civil-registration and cross-border systems, the absence of formal validation statistics for the matching process and the deterministic nature of the scenario modeling. The results therefore provide a strengthened surveillance baseline and a program-planning framework rather than causal evidence that re-engagement will produce the projected suppression gains.

Overall, this study demonstrates that systematic data cleaning is an essential component of HIV surveillance and program governance. Accurate denominators do not themselves improve clinical outcomes, but they are necessary for valid cascade measurement, appropriate resource allocation and identification of people who may benefit from renewed engagement in treatment and care.

## Limitations

This study has several limitations that should be considered when interpreting its findings.

### Reliance on historical routine data

The analysis relied on routinely collected clinical and program records that were not originally created for research. Some historical records contained incomplete identifiers, inconsistent treatment dates, missing transfer information and differences between paper and electronic documentation. Data cleaning reduced these inconsistencies but could not fully correct deficiencies in the original source records.

### Possible under-ascertainment of mortality

Although 1308 deaths were verified, mortality ascertainment may remain incomplete because the HIV registry was not fully interoperable with Guyana’s civil-registration and vital statistics system. Undocumented deaths may remain among individuals classified as long-term disengaged, particularly those from hinterland communities or without documented next-of-kin information.

The 10.2% classified as deceased represents accumulated historical mortality and should not be interpreted as a mortality rate because the records did not have a common follow-up period or defined person-time.

### Possible under-ascertainment of migration and silent transfer

Migration status was verified for 454 individuals. However, undocumented movement between Guyana, Suriname, Brazil, Venezuela and other countries may have resulted in residual misclassification. Some individuals classified as disengaged may have continued ART outside Guyana.

Internal facility transfers may also have been undocumented. Consequently, some patients classified as disengaged may have been receiving care at another facility within the national system.

### Potential errors in duplicate identification

The combination of unique-identifier matching, phonetic comparison and manual verification strengthened duplicate identification. Nevertheless, the sensitivity and specificity of the matching procedure were not formally evaluated. True duplicates may have remained undetected, while similar demographic profiles could have resulted in incorrect matches.

Inter-reviewer agreement was not measured, and the absence of a fully documented matching threshold may affect reproducibility.

### Absence of an explicit unverified category

All records were assigned to one of six outcome categories. The absence of a separate “unknown” or “outcome not verified” category may conceal residual uncertainty. Records without sufficient evidence of death, migration or continued treatment should be reported separately rather than being assigned a definitive status based only on their last recorded contact.

### Treatment-interruption definitions

The seven-year threshold used to separate short-term from long-term disengagement was operational rather than based on an established clinical standard. It may combine patients with substantially different probabilities of mortality, migration, silent transfer and successful re-engagement.

More granular intervals would provide greater clinical and programmatic value.

### Cross-sectional nature of the analysis

The study provided a validated snapshot of the registry but did not evaluate changes over time. It could not calculate annual treatment-interruption rates, time to disengagement, re-engagement incidence, mortality rates or predictors of treatment continuity.

Causal conclusions cannot be drawn regarding why patients disengaged or whether data cleaning itself improved patient outcomes.

### Limited sociodemographic and clinical variables

The dataset did not include sufficiently complete information on age, sex, gender identity, geographic location, key-population status, treatment regimen, viral-load history, drug resistance, pregnancy, stigma, mental health, transportation, income or other social determinants. The study therefore could not identify population groups experiencing disproportionate disengagement.

### Incomplete HIV cascade numerators

The study validated the treatment-eligible denominator but did not include complete verified numerators for the number receiving ART and the number with documented viral suppression. It was therefore unable to calculate definitive before-and-after estimates for the second and third UNAIDS 95 indicators.

The active-care proportion should not automatically be interpreted as ART coverage, and the modeling results represent potential changes in population-level suppression rather than direct recalculation of the third 95.

### Assumptions in viral-suppression modeling

The model assumed that 85% of successfully re-engaged individuals would achieve viral suppression. This may represent an optimistic scenario because successful outcomes depend on contactability, return to care, ART restart, adherence, drug resistance and continued retention.

The model did not incorporate uncertainty around these stages or undertake probabilistic sensitivity analysis. The percentage-point estimates should therefore be interpreted as scenario projections rather than predicted program outcomes.

### Limited generalizability

The findings are directly relevant to Guyana’s national HIV programme and may inform similar Caribbean settings. However, differences in population mobility, health-system organization, ART delivery and digital infrastructure may limit direct application to other countries.

## Recommendations

### Institutionalize national data validation

National HIV registry cleaning should be institutionalized as a routine process rather than conducted only as an occasional retrospective exercise. WHO recommends continuous assessment and improvement of HIV treatment and patient-monitoring data [2,9].

Guyana should:

• Conduct comprehensive national validation at least annually
• Perform quarterly facility-level reconciliation
• Establish a standardized national data-cleaning protocol
• Maintain an electronic audit trail for all corrected records
• Report data-quality indicators by facility and region

### Introduce a lifelong unique patient identifier

A confidential, lifelong identifier should be used across HIV testing, treatment, pharmacy, laboratory and referral services. The system should incorporate deterministic and probabilistic matching while retaining manual verification for uncertain matches.

Duplicate-detection algorithms should be formally validated by reporting sensitivity, specificity, positive predictive value and inter-reviewer agreement.

### Strengthen information-system interoperability

The HIV registry should be interoperable with:

• Civil registration and vital statistics
• Laboratory and viral-load systems
• Pharmacy and ART-dispensing records
• National electronic health records
• Hospital information systems
• Facility-transfer and referral systems

Interoperability would support timely identification of mortality, migration, silent transfer, treatment interruption and viral-load outcomes [2,12].

### Refine treatment-interruption categories

Treatment interruption should be classified using shorter, mutually exclusive intervals:

• 31-89 days
• 90-179 days
• 6-11 months
• 1-2 years
• More than 2 years
• Outcome unknown or unverified

These categories would enable the program to prioritize patients according to urgency, traceability and probability of successful re-engagement.

### Implement a tiered re-engagement strategy

The 1322 individuals with short-term treatment interruption should undergo outcome verification before tracing. Priority should be given to those with the most recent interruption, unsuppressed viral load, advanced HIV disease, pregnancy or other clinical vulnerabilities.

The re-engagement package should include:

• Confidential telephone and SMS contact
• Peer or patient navigation
• Community-based tracing where appropriate
• Rapid ART restart
• Multi-month medication dispensing
• Flexible appointments and community ART collection
• Transportation and psychosocial support

Evidence indicates that multi-component interventions can improve re-engagement, retention and viral suppression [13,14].

### Establish a re-engagement cascade

Program performance should be monitored through a standardized re-engagement cascade:

1. Identified as having interrupted treatment
2. Outcome verified
3. Confirmed alive and out of care
4. Successfully contacted
5. Returned to care
6. Restarted or continued ART
7. Retained at 3, 6 and 12 months
8. Viral-load test completed
9. Viral suppression achieved

This would distinguish failure to locate a patient from failure to return, remain in care or achieve suppression.

### Recalculate the national HIV cascade

Following linkage with pharmacy and viral-load systems, the program should recalculate:

• Percentage of diagnosed individuals receiving ART
• Percentage of individuals receiving ART with a documented viral-load result
• Percentage of individuals receiving ART who are virally suppressed
• Population-level viral suppression among all diagnosed individuals

The third UNAIDS 95 and population-level suppression should be reported separately.

### Strengthen the viral-suppression model

Future modeling should include:

• Conservative, moderate and optimal suppression assumptions
• Sensitivity analyses at approximately 56%, 70% and 85%
• Uncertainty around contact, return, ART restart and retention
• Confidence or simulation intervals
• Cost-effectiveness analysis
• Regional and facility-level scenarios

The projections should subsequently be compared with observed outcomes from a prospective re-engagement program.

### Conduct further research

Future research should:

• Identify demographic and clinical predictors of disengagement
• Examine geographic clustering of treatment interruption
• Evaluate reasons for disengagement through patient interviews
• Use survival analysis to assess time to interruption and re-engagement
• Evaluate the effectiveness of tracing and differentiated service delivery
• Assess drug resistance and viral suppression after ART restart
• Examine inequalities affecting key and vulnerable populations

### Protect confidentiality and data security

Digital integration and tracing must be accompanied by strong confidentiality protections. These should include role-based access, encryption, secure audit logs, minimum-necessary data access and confidential contact protocols.

Patients’ preferred methods and times of contact should be documented to reduce the risk of involuntary HIV-status disclosure.

### Priority implementation framework

**Table:** 

Priority action	Suggested timeframe	Performance indicator
Verify short-term interruption outcomes	0-6 months	Percentage with confirmed outcome
Launch targeted re-engagement	0-6 months	Percentage contacted and returned
Reconcile pharmacy and viral-load data	0-12 months	Percentage with linked records
Implement quarterly facility audits	Within 6 months	Facilities submitting validated data
Introduce refined interruption categories	Within 6 months	Records classified by duration
Link HIV registry with civil registration	6-18 months	Percentage of deaths electronically verified
Introduce a lifelong unique identifier	12-24 months	Percentage of records uniquely matched
Recalculate the national HIV cascade	Annually	Validated second- and third-95 indicators

## Conclusion

Systematic cleaning and validation of Guyana’s national HIV treatment and care registry reduced the historical registry from 12,824 records to a validated treatment-eligible population of 10,661 persons. The identification of 401 duplicate records, 1308 verified deaths and 454 confirmed migrations produced a total denominator correction of 16.9%. This finding demonstrates that unvalidated historical records can substantially affect national HIV programme denominators and the interpretation of treatment-cascade performance.

Within the validated cohort, 68.2% of persons were active in care, while 31.8% were classified as experiencing either short-term treatment interruption or long-term disengagement. The identification of 1322 persons experiencing short-term interruption provides a defined population for status verification and targeted re-engagement. However, this operational category remains heterogeneous and should be further stratified according to the duration of interruption.

The scenario analyses indicate that re-engagement could produce meaningful population-level viral-suppression gains, but the magnitude of the effect depends on successful return to care and subsequent suppression. As shown in Table 5, projected gains ranged from 1.0 to 6.3 percentage points across the re-engagement and suppression assumptions. These estimates are conditional planning scenarios rather than observed outcomes and should not be interpreted as a direct improvement in the third UNAIDS 95.

The principal contribution of this study is therefore the establishment of a more credible denominator for national HIV surveillance. Direct recalculation of the second and third UNAIDS 95 indicators will require linkage with complete, contemporaneous ART, pharmacy, laboratory and viral-load numerators. Institutionalizing routine registry validation, strengthening mortality and migration reconciliation, implementing lifelong unique identifiers and improving system interoperability will be essential to preserve these data-quality gains.

Overall, data cleaning should be recognized as a core public health and program-governance function. Accurate person-level information is necessary to measure the HIV response reliably, allocate resources appropriately and identify people who may benefit from renewed engagement in treatment and care. Prospective evaluation is now required to determine whether targeted re-engagement produces sustained ART retention and documented viral suppression in Guyana.

## CRediT authorship contribution statement

**Tariq Jagnarine:** Writing – review & editing, Writing – original draft, Visualization, Validation, Supervision, Software, Resources, Project administration, Methodology, Investigation, Funding acquisition, Formal analysis, Data curation, Conceptualization. **Murvin Chalmers:** Software, Data curation.

## Declaration of competing interest

The authors have no competing interests to declare.
